# Supplementary material for: Encapsulation of PI3K Inhibitor LY294002 within Polymer Nanoparticles Using Ion Pairing Flash Nanoprecipitation
Source: Pharmaceutics. 2023 Apr 6;15(4):1157. doi: 10.3390/pharmaceutics15041157 (PMC10145332; doi:10.3390/pharmaceutics15041157)
Supplement: Supplementary file 1 [file pharmaceutics-15-01157-s001.zip › pharmaceutics-2168695-supplementary.pdf]

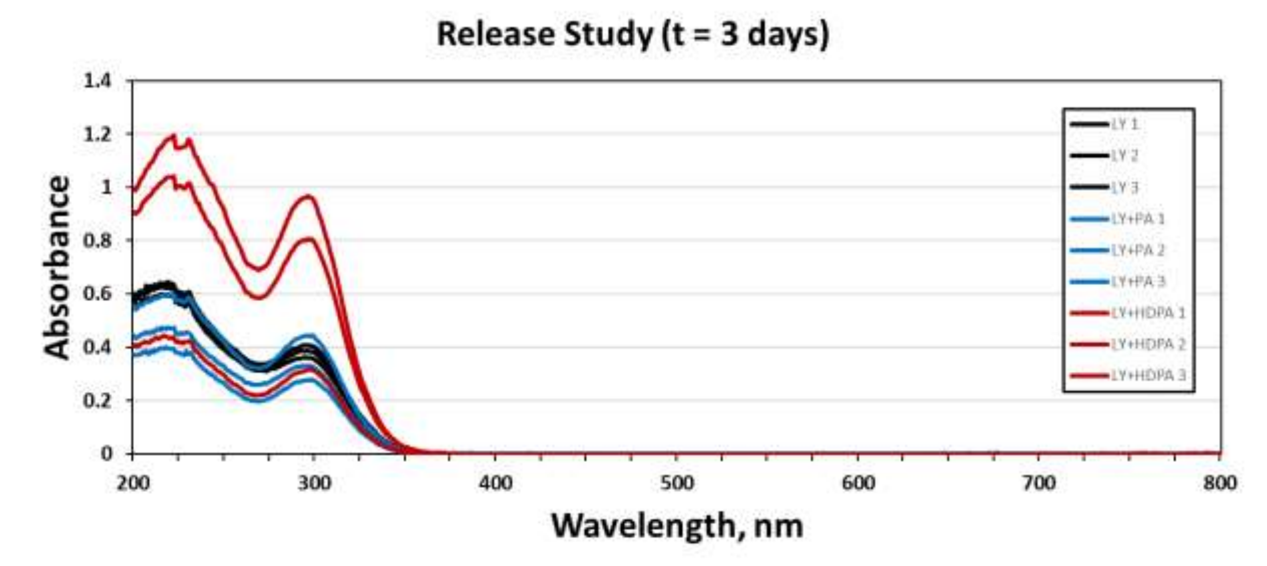

Figure S1. Exemplary UV-Vis absorbance spectra for LY294002 released from nanoparticles at the day 3 timepoint. Three replicates are shown for each tested case (i) LY only (black), (ii) nanoparticles containing a hydrophobic salt of LY and palmitic acid (blue), (iii) nanoparticles containing a hydrophobic salt of LY and hexadecyl phosphonic acid (red). Aliquots were taken from each diffusion cell and serially diluted prior to spectroscopy since the drug concentration was not known prior to measurement. The primary LY absorbance peak is found at a wavelength of 299 nm. For each replicate, the dilution with the highest concentration that yielded a curve within the linear absorbance regime at 299 nm (absorbance < 1) was selected to determine the amount of released LY. No detectable shift in the absorbance peak was observed for nanoparticles created with in situ ion pairing when compared to the drug only control nanoparticles. This suggests that the presence of an ion pairing agent does not influence the measured absorbance of LY during the release study. Additionally, absorbance values at longer wavelengths (> 400 nm) are negligible suggesting that the observed signal at 299 nm is not confounded with scattering from particles or aggregates.
